# Assessment of gadolinium deposition in the brain tissue of pediatric and adult congenital heart disease patients after contrast enhanced cardiovascular magnetic resonance

**DOI:** 10.1186/s12968-020-00676-2

**Published:** 2020-12-03

**Authors:** Neil Zaki, David Parra, Quinn Wells, Joshua D. Chew, Kristen George-Durrett, Sumit Pruthi, Jonathan Soslow

**Affiliations:** 1 Division of Pediatric Cardiology, Department of Pediatrics, Vanderbilt University Medical Center, Nashville, TN USA; 2 Departments of Medicine and Pharmacology, Vanderbilt University Medical Center, Nashville, TN USA; 3 Department of Radiology and Radiological Sciences, Vanderbilt University Medical Center, Nashville, TN USA

**Keywords:** Contrast enhanced MRI, CMR, Gadolinium, Gadolinium-based contrast agents

## Abstract

**Background:**

Contrast enhanced magnetic resonance imaging (MRI) is an important tool for the assessment of extracardiac vasculature and myocardial viability. Gadolinium (Gd) brain deposition after contrast enhanced MRI has recently been described and resulted in a warning issued by the United States Food and Drug Administration. However, the prevalence of brain deposition in children and adults with congenital heart disease (CHD) undergoing cardiovascular magnetic resonance (CMR) is unclear. We hypothesized that Gd exposure as part of one or more CMRs would lead to a low rate of brain deposition in pediatric and adult CHD patients.

**Methods:**

We queried our institutional electronic health record for all pediatric and adult CHD patients who underwent contrast enhanced CMR from 2005 to 2018 and had a subsequent brain MRI. Cases were age- and gender-matched to controls who were never exposed to Gd and underwent brain MRIs. The total number of contrast enhanced MRIs, type of Gd, and total Gd dose were determined. Brain MRIs were reviewed by a neuroradiologist for evidence of Gd deposition using qualitative and quantitative assessment. Quantitative assessment was performed using the dentate nucleus to pons signal intensity ratio (dp-SIR) on T1 weighted imaging. Continuous variables were analyzed using Mann–Whitney U and Spearman rank correlation tests. Normal SIR was defined as the 95% CI of the control population dp-SIR.

**Results:**

Sixty-two cases and 62 controls were identified. The most contrast enhanced MRIs in a single patient was five and the largest lifetime dose of Gd that any patient received was 0.75 mmol/kg. There was no significant difference in the mean dp-SIR of cases and controls (p = 0.11). The dp-SIR was not correlated with either the lifetime dose of Gd (r_s_ = 0.21, p = 0.11) or the lifetime number of contrast enhanced studies (r_s_ = 0.21, p = 0.11). Two cases and 2 controls had dp-SIRs above the upper bound of the 95% confidence interval for the control group. One case had qualitative imaging-based evidence of Gd deposition in the brain but had a dp-SIR within the normal range.

**Conclusion:**

In our cohort of pediatric and adult CHD patients undergoing contrast enhanced CMR, there was a low incidence of qualitative and no significant quantitative imaging-based evidence of Gd brain deposition.

## Introduction

Contrast enhanced cardiovascular magnetic resonance (CMR) is an important tool for the assessment of extracardiac vasculature and myocardial characterization. The use of gadolinium-based contrast agents (GBCAs) are often necessary to make an accurate diagnosis. Gadolinium (Gd) brain deposition after GBCA administration has been well-documented in both animal and post-mortem human studies [1–4]. Free Gd mimics ions such as calcium and zinc, causing direct toxicity to biologic systems [5, 6]. In a small number of case reports, Gd has been anecdotally linked to patients suffering from encephalopathy [7], recurrent pancreatitis [8], and acute tubular necrosis [9]. Aside from these acute reactions and the risk of nephrogenic systemic fibrosis, there has been no epidemiologic link between GBCA organ deposition and long-term risks to patient health. Nonetheless, concern about long-term Gd deposition recently resulted in a safety announcement and investigation by the United States Food and Drug Administration (FDA) regarding the use of GBCA [10] and self-imposed restrictions on GBCA use by neuroradiologists [11].

Brain magnetic resonance imaging (MRI) signal intensity (SI) has been noted to increase and remain elevated long after GBCA administration in adults [12]. Many studies have suggested that elevated brain MRI SI, particularly in the dentate nucleus, is associated with lifetime Gd dose [12–15]. Several pediatric studies have examined the effect of repeated GBCA administration on brain MRI SI in children with conflicting results [13, 15–24]. Some studies show that increased brain MRI SI is influenced by the chemical stability and amount of the specific GBCA used, while others show that SI is influenced by patients’ ages, underlying diagnoses, and the number of Gd studies they undergo. Of note, the study participants are most often patients with neurologic disease. Only one of the previously cited studies [17] considered the rate of Gd brain deposition in children without neurologic disease. No studies to date have considered the incidence of Gd brain deposition in pediatric and adult congenital heart disease (CHD) patients after contrast enhanced CMR.

CMRs often use a “double dose” or “one and a half times dose” of Gd (administration of 0.2 mmol/kg or 0.15 mmol/kg vs 0.1 mmol/kg) when assessing late gadolinium enhancement (LGE). Given the FDA’s safety communication and the theoretical long-term risk that this higher dose of Gd could have on patients, there is anecdotal evidence that clinicians in the fields of pediatric cardiology and adult CHD have restricted their use of GBCAs. The majority of studies, however, have included patients who received significantly higher lifetime doses of GBCAs. Unfortunately, there are no data to answer whether the limited use of contrast as part of a CMR in pediatric patients or adult CHD patients will place them at high risk of Gd brain deposition. To address this knowledge gap, we analyzed the brain imaging of pediatric and adult CHD patients who underwent at least one Gd enhanced CMR followed by a brain MRI. We hypothesized that Gd exposure as part of one or more CMRs would lead to a low rate of brain deposition in pediatric cardiology and adult CHD patients.

## Methods

### IRB

This study was approved by the Vanderbilt University Medical Center Institutional Review Board. Informed consent was waived because this was a retrospective study. All patient data were stored in a secure REDCap database [25].

### Study population selection

The study cohort was obtained by querying our CMR database for all patients who had undergone contrast enhanced CMR or contrast enhanced chest magnetic resonance angiography (MRA) from 2005 to 2018. This list of patients was then cross-referenced with a query of patients from the electronic health record (EHR) who had a CPT code for a brain MRI (Codes 7055, 170553, 70555, 70544, 70546, 76377, 70544, 70547, 70549, 70540, 70543, 70336). A detailed review of the remaining records was performed to identify which patients had undergone at least one brain MRI after the contrast enhanced CMR. If a patient had multiple brain MRIs or had MRIs conducted both prior to and after the CMR, then we included only the first and last brain MRIs in the analysis.

We collected study population demographic information including cardiac diagnosis, history of cancer, and history of inflammatory disease. We also collected information regarding age at CMR, age at brain MRI, indication for MRI, total number of contrast enhanced MRIs, and GBCA type and dose used with each MRI. When possible, we also recorded the serum blood urea nitrogen (BUN) and creatinine at the time of the contrast enhanced MRIs.

Patients frequently had both cardiac and non-cardiac contrast enhanced MRIs. Although our primary interest was in Gd deposition after CMR, we included all contrast enhanced MRIs in the analysis in order to assess lifetime Gd dose.

### Control population selection

A control population was determined by querying our institutional electronic health record to identify all pediatric (< 18 years of age) and adult (> 18 years of age) patients who underwent brain MRIs without contrast enhancement (CPT code 70551) from 2016–2018 and had no documented history of receiving Gd in the past. After excluding inadequate studies (see “Image analysis” section for exclusion criteria), the control population was matched one-to-one with the study population based on sex and age at the time of the study subject’s first brain MRI after CMR.

### Image analysis

CMRs were performed on a 1.5T CMR system (Intera, Philips Healthcare, Best, The Netherlands or Avanto, Siemens Healthineers, Erlangen, Germany). Brain MRIs were performed on 1.5T or 3T scanners (Philips Healthcare). All brain MRIs were reviewed by both an image analyst and neuro-radiologist in an unblinded manner using IMPAX (Agfa HealthCare, Mortsel, Belgium). Presence of axial T1-weighted images was confirmed by the readers and images were assessed for artifact or incidental findings that would preclude further study. Studies of acceptable quality were then analyzed by both the image analyst and neuroradiologist for both quantitative and qualitative evidence of T1 hyperintensity in the dentate nucleus. A subset of 29 studies was reviewed over 3 months later by the image analyst to assess intra-observer variability.

Quantitative signal intensity was determined using the same axial T1-weighted images. Signal intensity measurements were obtained by selecting a region of interest (ROI) in the dentate nucleus and the pons in the T1-weighted images. In each subject, ROI measurement was conducted once and always placed on the left side. If the left side could not be assessed, then the right side was used (n = 2).

Each ROI yielded an averaged signal intensity (SI) in that defined area. The SI of the dentate nucleus was divided by the SI of the pons, yielding the dentate nucleus-to-pons signal intensity ratio (dp-SIR). This method was used for all study and control brain MRIs. This method of determining SI ratio is similar to that used in other studies of Gd-related brain T1 hyperintensity [13, 15, 18, 24, 26].

### Statistical analysis

STATA (version 15; StataCorp LP, College Station, Texas, USA) was used to analyze the data obtained for this study. Statistical significance was set a priori to a p-value less than 0.05.

The dp-SIR upper-limit of normal was determined by calculating a 95% confidence interval from the control group’s mean dp-SIR. Students t-test was used to compare the difference in the dp-SI ratio between the study and control groups. Spearman rank correlation tests were calculated to determine if the study group dp-SIR correlated with lifetime dose of Gd (mmol/kg), lifetime number of contrast enhanced MRIs, age at time of CMR, and age at time of brain MRI. Subset analyses were performed using Mann–Whitney U tests to determine if there was a relationship between the type of GBCA received and dp-SIR, as many patients received more than one kind of contrast over their lifetimes. A Kruskal–Wallis test was used to compare dp-SIR to the underlying cardiac diagnoses of CHD, acquired heart disease, or no known heart disease and to evaluate for a difference between contrast agents (Magnevist (Bayer Healthcare, Berlin, Germany), Gadavist (Bayer Healthcare), and Omniscan (General Electric Healthcare)). Mann–Whitney U tests were used to compare dp-SIR to history of cancer and history of inflammatory disorders. A Wilcoxon signed-rank was used to compare the dp-SIR of patients who had brain MRIs both before and after contrast administration. Intra-observer variability was evaluated using an intraclass correlation coefficient and a Spearman rank correlation test.

## Results

### Baseline characteristics

We identified 302 patients who had undergone both CMR and brain MRI, 92 of whom underwent at least one contrast enhanced CMR prior to the brain MRI. Thirty patients had brain MRIs that were inadequate for analysis due to absence of axial T1 images or flow artifact in the region of the dentate nucleus or pons. Each of the remaining sixty-two patients had at least one brain MRI that was adequate for analysis and was performed after a contrast enhanced CMR. Their demographics and characteristics are summarized in Table 1. The comorbidities of cases and controls are summarized in Table 2.

**Table 1:** Characteristics of cases and controls

Characteristic	Cases (n = 62)	Controls (n = 62)
Male	50%	50%
Median age at first CMR (range)	14 years (4 days–51 years)	
Median age at first brain MRI after CMR (range)	15 years (18 days–54 years)	17 years (4 days–63 years)
Median number of contrast enhanced MRIs (range)	2 (1–5)	
Median lifetime dose of Gd (mmol/kg) (range)	0.28 (0.04–0.75)	0
Mean ± SD dp-SIR at first brain MRI after CMR	0.94 ± 0.08	0.92 ± 0.07

*CMR* cardiovascular magnetic resonance, *MRI* magnetic resonance imaging, *Gd* gadolinium, *dp-SIR* dentate nucleus-to-pons signal intensity ratio, *SD* standard deviation

**Table 2:** Comorbidities of cases and controls

	Cases n = 62	Controls n = 62
Cardiac diagnosis		
Congenital heart disease	35 (56%)	0
Acquired heart disease	11 (18%)	0
No known heart disease	16 (26%)	62
Cancer diagnosis	6 (10%)	0
Inflammatory disease diagnosis	16 (26%)	7 (11.3%)
Sickle cell disease	8	6
Inflammatory bowel disease	2	0
Other	6	1
Reasons for brain MRI		
Acute stroke concern	18 (29%)	11 (17.7%)
Chronic cerebrovascular disease	2 (3.2%)	3 (4.8%)
Headaches	11 (17.7%)	25 (40.3%)
Seizures	11 (17.7%)	8 (12.9%)
Sickle cell screening	6 (9.7%)	4 (6.5%)
Elevated ICP workup	6 (9.7%)	2 (3.2%)
Other	8 (12.9%)	9 (14.5%)

*MRI* magnetic resonance imaging, *ICP* intracranial pressure

There was no significant difference in age or gender between cases and controls. For cases, the median age at first brain MRI was 15 years (range 18 days to 54 years) and the median age at last brain MRI was 17 years (range 4 days to 63 years). The median time between CMR and brain MRI was 2.6 years (range 3 days to 11.4 years). The CMR was the last contrast enhanced MR prior to the brain MRI in all but 3 patients. Thus, the median time from any contrast enhanced MRI to brain MRI was 2.5 years (range 3 days to 11.4 years).

The number and type of contrast enhanced MRIs that patients underwent prior to the last brain MRI in our database varied. Eighteen patients (29%) had 2 contrast enhanced CMRs and 3 patients (5%) underwent 3 contrast enhanced CMRs. Twenty-one patients (34%) in the study population also underwent at least one non-cardiac contrast enhanced MRI prior to the last brain MRI (range 1–4 MRs). The total number of contrast enhanced MRIs (CMR + non-cardiac MRI) that patients underwent was 1 to 5 with a median of 2 per patient. The amount of contrast received in a single study ranged from 0.04 mmol/kg to 0.3 mmol/kg. The lifetime range of contrast received ranged from 0.04 to 0.75 mmol/kg with a median of 0.21 mmol/kg. The linear ionic agent gadopentetate (Magnevist, Bayer HealthCare) was the contrast agent used in the majority of CMRs (n = 62, 73%) and non-cardiac MRIs (n = 18, 53%). The macrocyclic agent gadobutrol (Gadavist, Bayer HealthCare) was used in 19% (n = 11) of CMRs and 33% (n = 12) of non-cardiac MRIs (this change represented an institution-wide move to use of gadobutrol). A smaller number of studies used the linear nonionic agent gadodiamide (Omniscan, General Electric Healthcare) or the linear ionic agents gadobenate (MultiHance, Bracco Diagnostics, Milan, Italy), gadoxetate (Eovist, Bayer HealthCare Pharmaceuticals), and gadofosveset (Ablavar, Lantheus Medical Imaging, North Billerica, Massachusetts, USA). There were 2 studies in which the available EHR documentation did not specify the type of contrast used.

### Determining normal SI ratio ranges

**Fig. 1:**
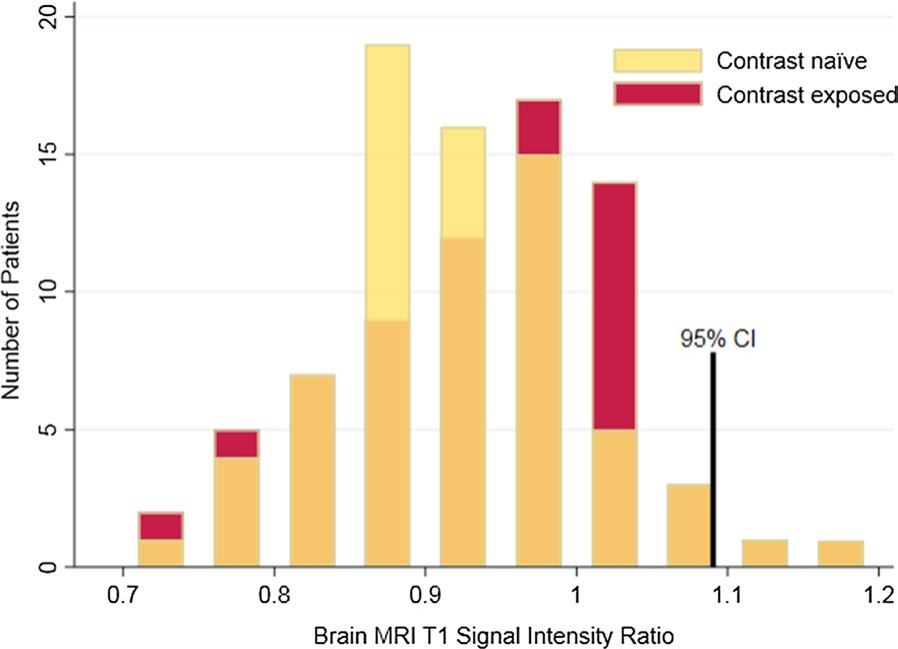
Distribution of brain magnetic resonance imaging (MRI) T1 signal intensity ratio for those exposed to contrast (cases, red) and those who are contrast naïve (controls, yellow). The overlap of these two histograms is represented with orange

**Fig. 2:**
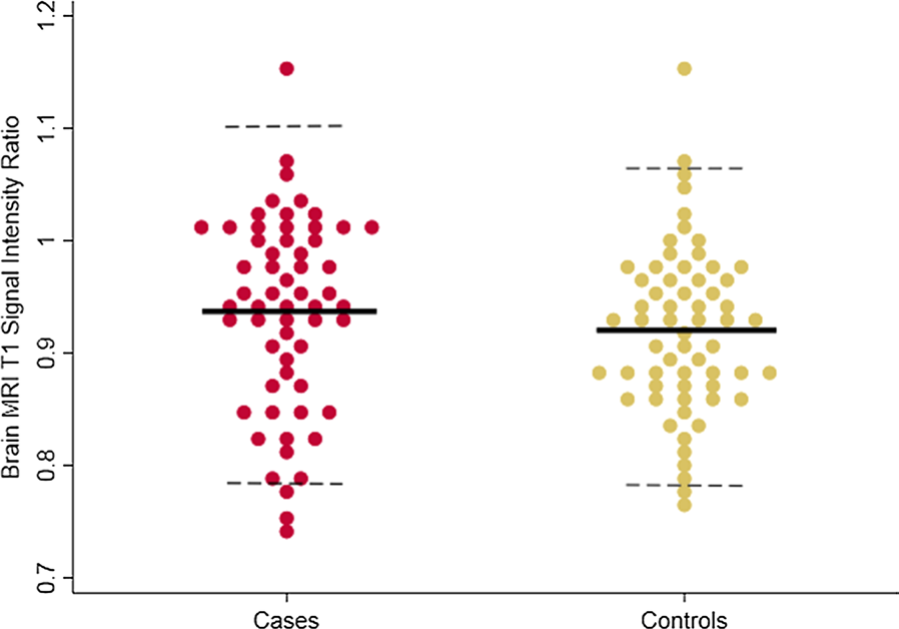
Dot plot comparison of signal intensity ratio distribution between cases and controls. Solid line indicates mean, dotted lines indicate 95% CI

A histogram was generated to evaluate the distribution of the control and study dp-SIRs (Fig. 1). Cases and controls did not have significantly different means (0.94 ± 0.08 vs 0.92 ± 0.07, p = 0.11) (Fig. 2). There was strong intra-observer agreement of brain MRI SI measurements, with an intraclass correlation coefficient of 0.75 (p < 0.001) and a Spearman correlation between measures of 0.76 (p < 0.001).

### Signal intensity analysis

The dp-SIR ranged from 0.76–1.15 with a mean of 0.92 (95% CI 0.79, 1.04) for controls and from 0.75–1.15 with a mean of 0.94 (95% CI 0.781, 1.07) for cases (Fig. 1). Based on our pre-defined upper limit of normal as the upper bound of the 95% CI of the control group, there were 4 subjects [2 controls, 2 cases] with abnormal dp-SIR. None of these individuals had qualitative imaging-based evidence of brain Gd deposition. The two cases had both undergone two contrast enhanced MRIs prior to the last brain MRI.

There was one individual in the study population, whose dp-SIR (0.99) was within the determined normal range but had qualitative imaging-based evidence of brain Gd deposition. There were no individuals in the control group with qualitative imaging-based evidence of brain Gd deposition.

### Correlation of SIR and study-population characteristics

**Fig. 3:**
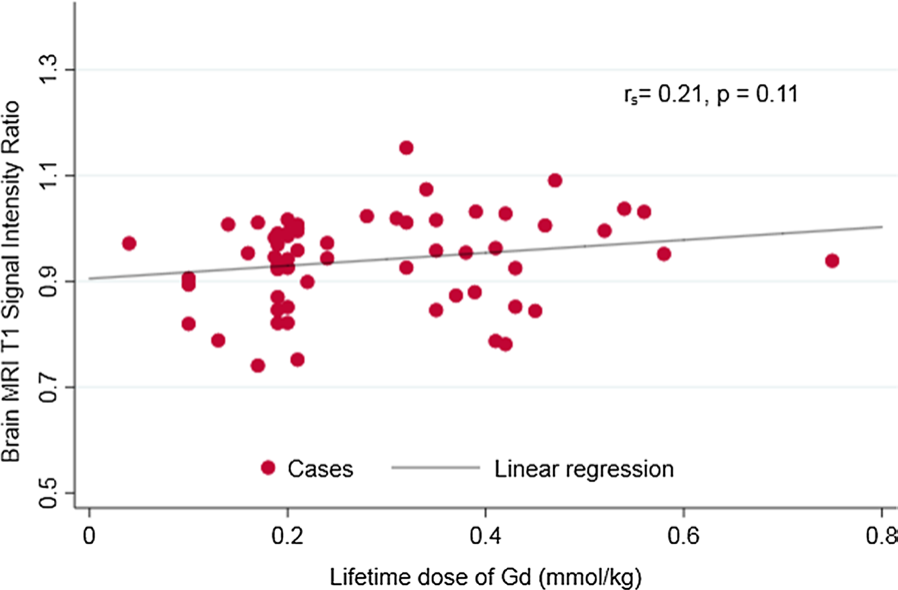
Relationship of lifetime gadolinium (Gd) dose to brain MRI T1 signal intensity ratio. Gd, *gadolinium*

**Fig. 4:**
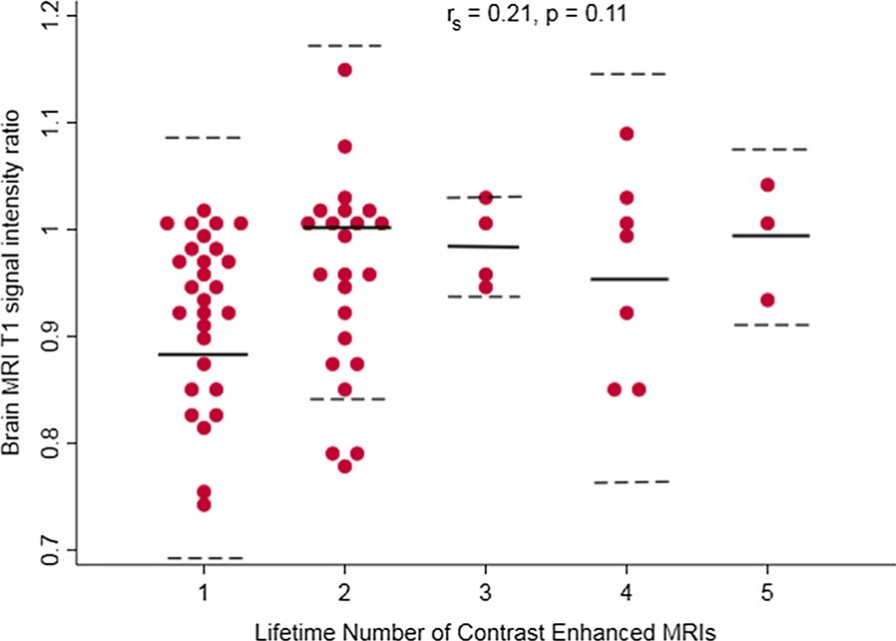
Relationship between number of contrast enhanced MRIs and brain MRI T1 dentate nucleus to pons signal intensity ratio. Solid lines represent mean signal intensity ratio. Dashed lines represent 95% CI

The dp-SIR measured from the first brain MRI after CMR did not correlate with the lifetime dose of Gd (r_s_ = 0.21, p = 0.11) (Fig. 3). The lifetime number of contrast enhanced studies also did not correlate with dp-SIR (r_s_ = 0.21, p = 0.11) (Fig. 4). There was no difference in mean values of dp-SIR in patients who received the linear nonionic agent gadopentetate (Magnevist), the macrocyclic nonionic agent gadobutrol (Gadavist), or linear ionic agent gadodiamide (Omniscan), p = 0.37. Subset analysis demonstrated no correlation of dp-SIR with exposure to gadopentetate (n = 50, p = 0.64), gadobutrol (n = 16, p = 0.86), or gadodiamide (n = 7, p = 0.94).

The dp-SIR is weakly correlated with patient age at first brain MRI in cases (r_s_ = 0.38, p = 0.003) and controls (r_s_ = 0.42, p < 0.001). The dp-SIR was not significantly different based on patient sex (p = 0.77), history of cancer (p = 0.11), or history of other inflammatory disorders (p = 0.07). The dp-SIR did not differ between the types of heart disease (CHD, acquired heart disease, or no known heart disease) (p = 0.33).

**Fig. 5:**
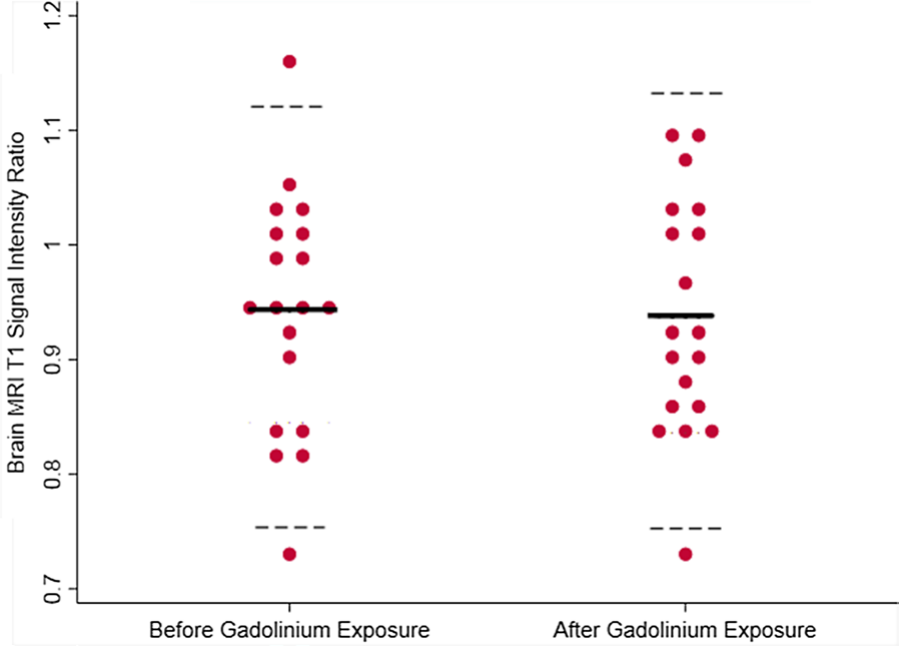
The range of dentate nucleus to pons signal intensity ratios in those patients who had brain MRI both before and after receiving gadolinium-based contrast. Solid line represents the mean signal intensity ratio. Dashed lines represent 95% CI

Nineteen patients had multiple brain MRIs both before and after contrast enhanced CMR. The dp-SIR of the brain MRI before CMR did not differ from the dp-SIR of the last brain MRI after CMR (p = 0.90) (Fig. 5).

## Discussion

Our study is, to our knowledge, the first to evaluate evidence of Gd deposition in the brains of patients after contrast enhanced CMR. We found that there is no significant difference between the dp-SIR of patients who have undergone contrast enhanced CMR and those who have never received a GBCA. The dp-SIR in our patient population is not significantly associated with type of heart disease, history of inflammatory disorders, history of cancer, patient sex, lifetime amount of contrast exposure, number of contrast enhanced MRIs, or type of contrast used. The dp-SIR does correlate with age in cases and controls.

Our image analysis methods are similar to those used in other studies of brain MRI SI after GBCA administration [13, 15, 18, 24, 26]. Our analysis, however, of GBCA exposure and brain MRI SIRs differs from prior work in two key ways. First, our patients were exposed to lower lifetime doses of Gd than patients in similar studies and drastically lower doses than in animal-based studies [1]. The most contrast enhanced MRIs that any of our patients underwent was five, which was often the minimum number required to meet inclusion criteria in other studies [15, 22, 26]. Nonetheless, some have found that even in adults with more than 20 Gd administrations [17], there was no difference between the dp-SIR of patients who received Gd and those who had never been exposed to GBCAs.

Second, our patients’ comorbidities differ from prior investigations. Most prior studies that show an association between GBCA administration and increased brain MRI SI were conducted in patients with neurologic disease, such as intracranial neoplasms. It is possible that inflammation or a primary brain neoplasm increases permeability of the blood–brain barrier, thus increasing the risk that GBCAs may cross and be deposited in the brain. Furthermore, many factors beyond just GBCA exposure have been associated with increased brain MRI SI, including radiation therapy, hepatic dysfunction, Wilson’s disease, Rendu–Osler–Weber syndrome, manganese toxicity (as from parenteral nutrition), calcifications, hemodialysis, and neurofibromatosis type 1 [12, 27, 28]. As most of our patients were children or young adults, they tended to have fewer comorbidities that may have increased their brain MRI SI.

The relationship between age and T1-weighted brain MRI signal intensity has been documented [29–32], though its reason is unknown. Similarly, the normal rate of increase in signal intensity is also unknown. To account for this relationship, we age-matched our cases and controls. As older patients are more likely to have received a higher lifetime dose of Gd, this is a confounding variable in any study of the relationship between Gd and brain MRI signal intensity.

## Study limitations

We acknowledge that there are several limitations to the present study. There is a possibility of type II error. Based on the standard deviation we found in this study (0.08), however, we performed a power calculation to determine the detectable alternative with our current sample size and we were powered to detect a difference as small as ± 0.041. While there may still be a statistically significant difference between the cohorts, 0.041 represents a very small difference that is not likely to be clinically significant.

As a retrospective study, we were unable to randomize patients or otherwise control for confounding variables. This is a single center study which may decrease generalizability. Only a minority of patients had more than 3 contrast enhanced MRIs and only two patients had 5 contrast enhanced studies. Even in patients with complex CHD, however, it is uncommon to undergo more than 5 contrast enhanced CMRs. Without records from other hospitals, we cannot exclude the possibility that patients underwent imaging elsewhere before admission to our institution. Thus, the number of exposures to GBCAs may have been underestimated and “control” patients may have had prior GBCA exposure. Interpretation of SI can be subjective and small changes in the boundaries of an ROI can create relatively large differences in SI [17]. We minimized variability by having one image analyst analyze all images and having a neuroradiologist confirm all subjective assessments and ROIs.

An additional limitation of the present study is the use of T1 hyperintensity as a surrogate for Gd deposition. A lack of T1 hyperintensity may belie anatomic evidence of Gd deposition on autopsy. Additionally, we cannot exclude the possibility that deposited Gd may be slowly cleared from the brain, which could confound measurements in those patients with an extended period between the last contrast enhanced MRI and the last brain MRI.

## Conclusions

Pediatric and adult CHD patients who undergo contrast enhanced CMR have no significant difference in dp-SIR when compared with controls. Only one patient had qualitative evidence of Gd brain deposition, and this patient had a normal dp-SIR. While we agree with limiting exposure to Gd contrast whenever possible, our data suggest that, when clinically indicated, GBCAs with CMR are unlikely to lead to significant brain deposition in this population.

## Data Availability

The datasets generated and analyzed during the current study are not publicly available due to the primary dataset’s inclusion of protected health information but some data are available from the corresponding author on reasonable request. In accordance with our institutional review board policies, these datasets will remain available for 2 years following publication, after which time they will be destroyed.

## Abbreviations

CHD: Congenital heart disease
CMR: Cardiovascular magnetic resonance
dp-SIR: Dentate nucleus-to-pons signal intensity ratio
FDA: United States Food and Drug Administration
Gd: Gadolinium
GBCA: Gadolinium-based contrast agent
ICP: Intracranial pressure
LGE: Late gadolinium enhancement
MRA: Magnetic resonance angiography
MRI: Magnetic resonance imaging
ROI: Region of Interest
SI: Signal intensity
